# Sclerosing angiomatoid nodular transformation of the spleen in a patient with Maffucci syndrome: a case report and review of literature

**DOI:** 10.1186/s13000-017-0670-z

**Published:** 2017-11-10

**Authors:** Xiao-Dan Huang, Hao-Sen Jiao, Zheng Yang, Chuang-Qi Chen, Yu-Long He, Xin-Hua Zhang

**Affiliations:** 1 Zhongshan School of Medicine, Sun Yat-sen University, 74 Zhongshan Second Road, Guangzhou, China; 2 Department of Pathology, The First Affiliated Hospital of Sun Yat-Sen University, 58 Zhongshan Second Road, Guangzhou, China; 3 Department of Gastrointestinal Surgery, The First Affiliated Hospital of Sun Yat-Sen University, 58 Zhongshan Second Road, Guangzhou, 510080 China

**Keywords:** Spleen, Maffucci syndrome, SANT, Visceral vascular lesions

## Abstract

**Background:**

Maffucci syndrome is a congenital, non-hereditary mesodermal dysplasia characterized by multiple enchondromas and hemangiomas. The presence of visceral vascular lesions in this syndrome is exceedingly rare.

**Case presentation:**

We report a 26-year-old female who was diagnosed with Maffucci syndrome along with sclerosing angiomatoid nodular transformation (SANT) of the spleen. The patient underwent a laparoscopic splenectomy. Immunostaining of the excised specimen revealed 3 distinct types of vessels in the angiomatoid nodules: CD34−/CD8−/CD31+ small veins, CD34−/CD8+/CD31+ sinusoids, and CD34+/CD8−/CD31+ capillaries, leading to the diagnosis of SANT of the spleen.

**Conclusions:**

This case reports the first patient in the literature exhibiting the features of Maffucci syndrome along with SANT of the spleen. The spleen is probably a predilection site of visceral vascular lesions in this syndrome with a proportion of 4 out of 14. An abdominal Computed Tomography (CT) scan is recommended for any cases of abdominal discomfort. Surgical excision is usually sufficient because of the relatively benign behavior of SANT, however, a more aggressive follow-up is proposed due to the high risk of malignant transformation of enchondromas and development of other neoplasms associated with this syndrome. Further studies are required to reveal its genetic basis for comprehensive prognosis evaluation and therapeutic guidance.

## Background

Maffucci syndrome, first described by Angelo Maffucci in 1881, is a congenital, non-hereditary mesodermal dysplasia characterized by multiple enchondromas and hemangiomas [1]. A variation of this syndrome with the presence of visceral vascular lesions is exceedingly rare as most of the lesions manifest themselves in subcutaneous areas. So far, approximately 200 cases have been reported world-wide and only 13 involve the non-cutaneous vascular lesions [2–14]. Hence the purpose of this report is to present a case of Maffucci syndrome associated with SANT, a nonneoplastic vascular lesion of the spleen, together with a review of the available English and Chinese literature. To the best of our knowledge, this is the first documented case of SANT in spleen in the context of Maffucci syndrome. We have obtained the patient’s informed consent allowing us to print and electronically publish this case report.

## Case presentation

A 26-year-old Chinese female was admitted to the First Affiliated Hospital of Sun Yat-Sen University with recurrent abdominal pain for 4 years and splenomegaly for 1 year. Four years ago, she began to suffer from dull recurrent upper abdominal pain, especially while sleeping at night. The pain gradually became more localized to the left subcostal region where an abdominal mass (measuring about 2.0 × 1.5 cm) could be palpable for 1 year. The patient complained that the mass enlarged, with a new measurement of about 4.0 × 3.0 cm, and was accompanied by moderately aggravated pain especially during deep breath over the past month. The mass was later confirmed to be the enlarged spleen by abdominal ultrasonography. Since disease onset she experienced nausea and retching, and recently, sporadic headache and left periorbital pricking and melena. The patient had a past history of excision of two bony lumps of the left ankle and the right knee at the age of 16 and 18 respectively without post-excision detailed pathological classification.

**Fig. 1:**
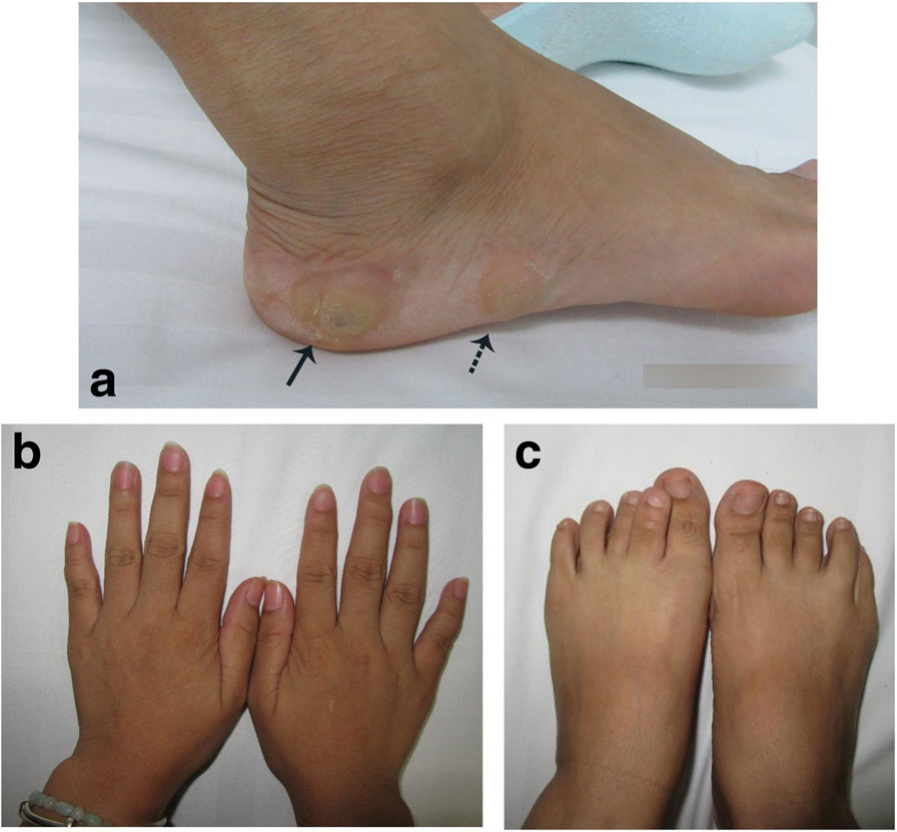
The gross pictures of the patient. See two skin-colored nodules (arrows) on lateral side of the right foot (**a**). Note that the bigger one (solid arrow) shows a little blue in color. The patient’s fingers (**b**) and toes (**c**) were deformed due to enchondromas

Physical examination revealed that the spleen was palpable 3 cm subcostal on the left anterior axilllary line with solid texture, dull-smooth margin and mild tenderness. In addition, there were two tender soft-tissue masses, perhaps venous malformation, on the lateral side of the right foot (Fig. 1a) Apart from those, firm multiple nodular lesions were found on the extremities, especially on the fingers and toes (Fig. 1b and c). The patient informed us that the bony lesions of the hand appeared more than 10 years ago.

**Fig. 2:**
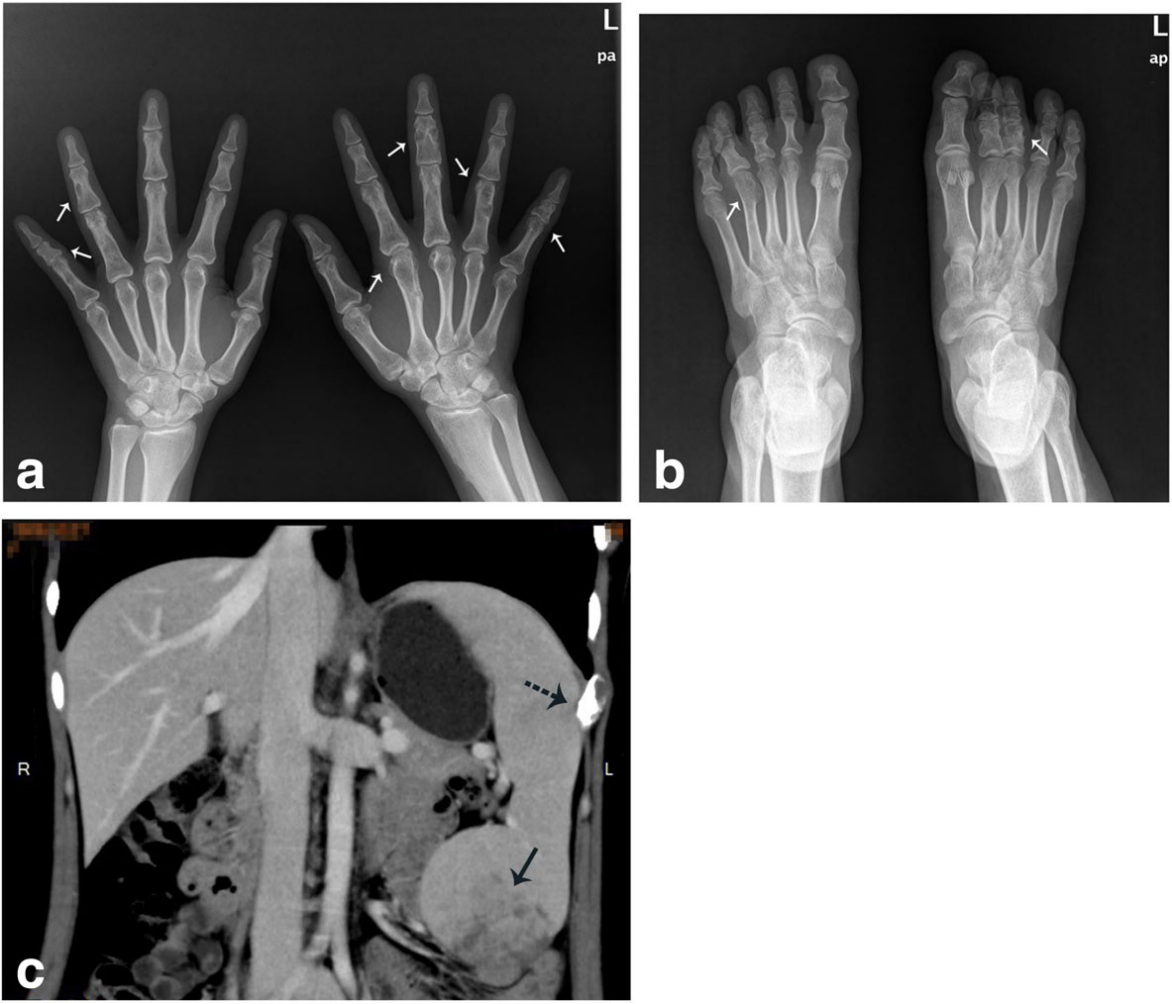
Preoperative imaging studies. CR images of both hands (**a**) and feet (**b**) show multiple bone deformations (white arrows). The abdominal CT (**c**) demonstrates a huge nodular hyperplasia of the spleen which is considered as angioma (black solid arrow), and a vascular lesion of the 10th rib (black doted arrow)

Radiography revealed irregularly shaped radiolucent areas with stippled calcification within the tubular bones of both hands and feet, which were diagnosed as enchondromas (Fig. 2a and b). CT scan of the upper abdomen demonstrated nodular hyperplasia of the spleen, most likely considered as benign angioma, and some vascular lesions of the left 8th~10th ribs, suspected as hemangiomas, were found as well (Fig. 2c). Gastroscopy discovered upper-esophageal venous angioma measuring 0.5 × 0.7 cm. Cranial CT showed no abnormalities. Based on these findings, the development of multiple enchondromas in the extremities and the presence of both subcutaneous and visceral vascular lesions, the diagnosis of Maffucci syndrome was established.

**Fig. 3:**
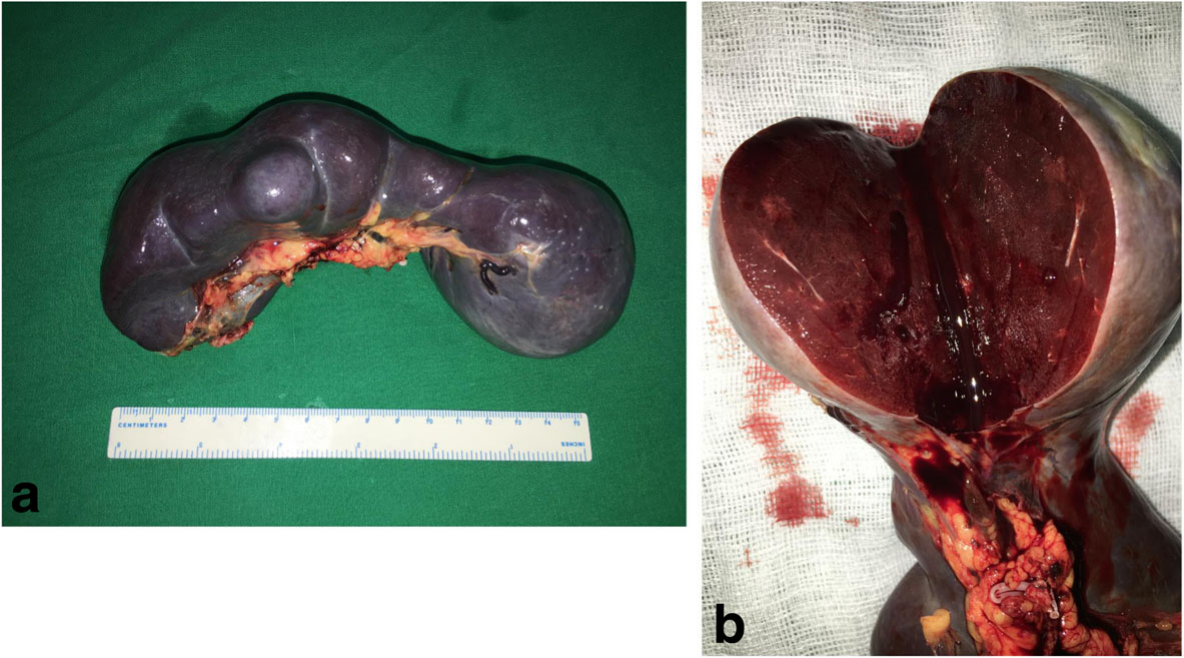
The gross pictures of the excised spleen. The excised spleen presents with a multinodular appearance (**a**) and the excised surface is red-brown in color (**b**)

The patient underwent a laparoscopic splenectomy. Macroscopically, the excised specimen was measured 14 × 6 × 6 cm, with the excised surface displaying a mass of coalescing red-brown nodules embedded in a dense fibrous stroma (Fig. 3).

**Fig. 4:**
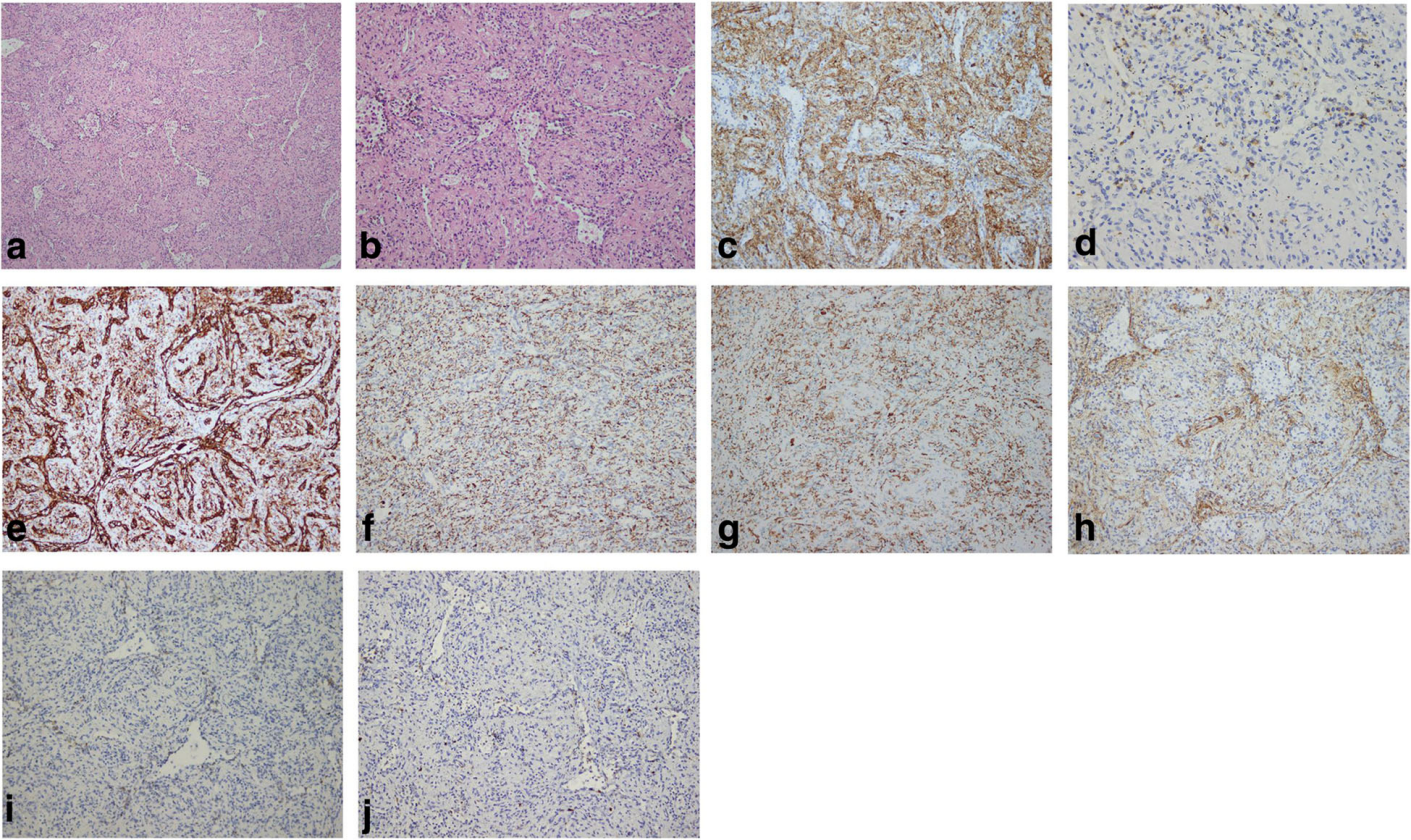
Microscopical features and immunohistochemistry of the pathological sections of the spleen. The Hematoxylin-Eosin staining ((**a**) H&E staining ×2; (**b**) H&E staining, ×200) shows multiple small nodules formed by proliferative spindle cells without obvious cellular atypia or mitotic figures. The immunostaining revealed 3 distinct types of vessels in the angiomatoid nodules: CD34−/CD8−/CD31+ small veins, CD34−/CD8+/CD31+ sinusoids, and CD34+/CD8−/CD31+ capillaries ((**c**) CD34 × 200; (**d**) CD8 × 200; (**e**)CD31 × 200). Some endothelial cells expressed CD68 while lacking staining of actin, desmin, S-100 and CD163 ((**f**) CD68 × 200; (**g**) CD163 × 200; (**h**) Actin; (**i**) Desmin; (**j**) S-100)

Microscopically, multifocal ectopic granulomatous reaction and numerous red blood cells were presented, and a mass of coalescing small nodules were composed of slit-like, round or irregular-shaped vascular spaces lined by plump endothelial cells and interspersed by a population of spindly or ovoid cells. Nuclear atypia was minimal, mitotic figures were extremely rare, and necrosis was consistently absent (Fig. 4a and b). Immunostaining revealed 3 distinct types of vessels in the angiomatoid nodules: CD34−/CD8−/CD31+ small veins, CD34−/CD8+/CD31+ sinusoids, and CD34+/CD8−/CD31+ capillaries (Fig. 4c-e). Furthermore, some endothelial cells expressed CD68 while lacking staining of actin, desmin, S-100 and CD163 (Fig. 4f-j). Based on these findings, the diagnosis of sclerosing angiomatoid nodular transformation (SANT) of the spleen was rendered.

## Discussion and conclusions

Maffucci syndrome bears no sex or race predominance and is a rare, congenital condition which usually presents early in life. It typically manifests as the coexistence of enchondromas with vascular lesions, distinguished from Ollier’s disease with only the presence of enchondromas in the latter [1].

Enchondromas usually develop in close proximity to growth plate cartilage as a potential result from deregulated proliferation and differentiation of chondrocytes during physiological enchondral ossification [15]. They are commonly in the small bones of fingers and toes, as in our case, long tubular bones, and flat bones such as the pelvis. The tendency for malignant transformation of enchondromas into chondrosarcomas is a well-known fact. According to a retrospective multicenter study by Verdegaal.et al. [16], overall incidence of development of chondrosarcomas is 40%. Furthermore, Schwartz et al. [17] reported that malignant degeneration was almost a certainty for patients with Maffucci syndrome. In view of the poor prognosis associated with malignant transformation, patients should be under life-long surveillance for early detection of occult malignant lesions.

Vascular lesions associated with this syndrome usually present as unilateral subcutaneous hemagiomas, the blue nodules that can be emptied by manual compression. It is noticed that in our case the nodules are not so blue (Fig. 1a). We suppose that this may be an early manifestation and perhaps they will eventually turn blue. Combined occurrence of visceral lesions is exceptionally rare while this case presentation involves the spleen. With all the available English and Chinese literature reviewed, we found only 13 other cases had been described (Table 1) and 3 of them also involved the spleen [2, 4, 14]. Combined with this case, the incidence of the spleen involvement was approximately 28.6% (4/14), indicating that the spleen is likely to be a predilection site of visceral vascular lesions in Maffucci syndrome. Therefore, we recommend an additional abdominal CT scan following the diagnosis of Maffucci syndrome.

**Table 1:** Cases of Maffucci syndrome with vascular lesions presenting in non-cutaneous regions

References	Age(year)/sex	Location	Pathology	Follow up	Year
Present report	27/F	Spleen	SANT	AWD 8 months	2017
Lin et al. [12]	39/M	Small intestine	NA	NA	2015
Yu Cai et al. [3]	34/F	Lower lip	Venous malformation and epithelioid hemangioma	NA	2013
Pezzilli et al. [10]	51/F	Liver	Hepatic hemangioma	AWD 1 month	2009
Lotfi et al. [8]	23/M	Tougue	Cavernous hemangiomas	AWD 1 year	2009
Lee et al. [7]	10/F	Hypopharynx and ascending colon	NA	NA	1999
Ahmed et al. [2]	29/F	Spleen	NA	NA	1999
Fanburg et al. [4]	13/M	Spleen	Low-grade angiosarcoma	AWD 18 years	1995
Wolf et al. [13]	7/M	Tonsils	Cavernous hemangiomas	AWD 1 year	1993
Zhang et al. [14]	13/F	Spleen	Cavernous hemangiomas	NA	1990
Laskaris et al. [6]	24/M	Tongue	Cavernous hemangiomas	AWD 3 years	1984
Lowell et al. [9]	27/F	Hypopharynx, pharynx	Cavernous hemangiomas	AWD 4 years	1979
Hall et al. [5]	30/M	Rectosigmoid	None	AWD 15 years	1972
Sakurane et al. [11]	31/F	Hypopharynx, esophagus, anal, ileum and lower lip	Cavernous hemangiomas	AWD 1 year	1967

*AWD* alive with disease, *F* female, *M* male, *NA* not available, *SANT* sclerosing angiomatoid nodular transformation

It has previously been demonstrated that three types of vascular lesions--cavernous hemangiomas, phlebectasias and lymphangiectasias ± lymphangiomas, are associated with Maffucci syndrome [7]. However, some of the cavernous hemangiomas are actually spindle cell hemangiomas (SCH), which are much more specific for this syndrome [1, 18]. The pathology of those 3 cases with splenic lesions are respectively hemangioma (without further histological classification) [2], low-grade angiosarcoma (the only malignant one) [4] and cavernous hemolymphangioma (reported in the Chinese literature) [14],while our case presents as SANT, a nonneoplastic vascular lesion of spleen. To our knowledge, it is the first case of this pathological type to be described in Maffucci syndrome. With the distinctive architectural features, SANT is composed of different specialized types of blood vessels characteristic of the splenic red pulp rather than a single type as in hemangioma, probably representing a peculiar transformation of the red pulp in response to an exaggerated stromal proliferation rather than a primitive level of vascular differentiation [19].

Reported vascular lesions have seldomly undergone malignant transformation and only one with spleen involvement mentioned above represents a debatable example of malignant transformation of SCH [4, 18]. In this case, SANT is a relatively benign lesion characterized by the low proliferative index, lack of cytologic atypia, and uniformly benign clinical evolution, and splenectomy has so far proved curative in every instance [19].

According to the current literature, patients with Maffucci syndrome are at increased risk of developing different kinds of malignant tumors, including intracranial tumors, hepatic carcinoma and so on [1, 16]. Since our patient had some neurological complaints, a cranial CT was given and although there was no abnormal findings, she deserves reexamination in case of any neurological symptom exacerbations. In addition, her complaint of melena might be simply related to the upper-esophageal venous angioma discovered by gastroscopy but further examination and regular follow-up are still recommended to detect any other more severe gastrointestinal lesions, as commonly seen in other cases [5, 7, 11, 12].

The genetic basis of this syndrome is still unclear partly due to its lack of familial transmission, but recently it has been connected to somatic mutations in the genes coding for the cytoplasmic and mitochondrial isoforms of isocitrate dehydrogenase, IDH1 and IDH2 [1, 15, 20, 21]. Pansuriya et al. found that there are also mutations of IDH1 and IDH2 in enchondromas and spindle cell hemangiomas and 77% of patients with Maffucci syndrome carried IDH1 (98%) and IDH2 (2%) mutations [21]. The fact that no genetic transmission of this syndrome has ever been observed would fit the notion of somatic mosaicism-germline heterozygosity being presumably incompatible with life [15]. Furthermore, heterozygous somatic mutations in IDH1/IDH2 are also associated with other various tumor types, including glioma, glioblastoma, acute myeloid leukemia, and intrahepatic cholangiocarcinomas [22], which may explain the carcinogenesis in this syndrome. But since our patient declined any further genetic tests in view of its relatively high expense and currently unknown benefit for therapeutic guidance, we have no idea whether she carries the same mutation and can’t offer any more specific evaluation on her prognosis concerning malignant transformation. Further studies are required of patients with Maffucci syndrome not only to research into its etiology but also with respect to its comprehensive prognosis evaluation and therapeutic decision.

## Abbreviations

CT: Computed Tomography
SANT: Sclerosing angiomatoid nodular transformation
SCH: Spindle cell hemangiomas
